# NRF2 and Bip Interconnection Mediates Resistance to the Organometallic Ruthenium-Cymene Bisdemethoxycurcumin Complex Cytotoxicity in Colon Cancer Cells

**DOI:** 10.3390/biomedicines11020593

**Published:** 2023-02-16

**Authors:** Alessia Garufi, Riccardo Pettinari, Fabio Marchetti, Mara Cirone, Gabriella D’Orazi

**Affiliations:** 1 Department of Research and Advanced Technologies, IRCCS Regina Elena National Cancer Institute, 00144 Rome, Italy; 2 Chemistry Interdisciplinary Project (CHIP), School of Pharmacy, University of Camerino, 62032 Camerino, Italy; 3 Chemistry Interdisciplinary Project (CHIP), School of Science and Technology, University of Camerino, 62032 Camerino, Italy; 4 Laboratory Affiliated to Pasteur Institute Italy Foundation Cenci Bolognetti, Department of Experimental Medicine, Sapienza University of Rome, 00161 Rome, Italy; 5 Department of Neurosciences, Imaging and Clinical Sciences, University “G. D’Annunzio”, 66013 Chieti, Italy; 6 School of Medicine, UniCamillus International University of Health Sciences, 00100 Rome, Italy

**Keywords:** colon cancer, curcumin, ruthenium-cymene complex, cell death, unfolded protein response, BiP, CHOP, NRF2, DNA damage, chemoresistance

## Abstract

Organometallic ruthenium (Ru)(II)-cymene complexes display promising pharmacological properties and might represent alternative therapeutic agents in medical applications. Polyphenols, such as curcumin and curcuminoids, display beneficial properties in medicine, including chemoprevention. Here we analyzed the anticancer effect of a cationic Ruthenium (Ru)(II)-cymene Bisdemethoxycurcumin (Ru-bdcurc) complex. The experimental data show that Ru-bdcurc induced cell death of colon cancer cells in vitro. In response to treatment, cancer cells activated the endoplasmic reticulum (ER)-resident chaperone GRP78/BiP and NRF2, the master regulators of the unfolded protein response (UPR) and the antioxidant response, respectively. Pharmacologic targeting of either NRF2 or BiP potentiated the cytotoxic effect of Ru-bdcurc. We also found that NRF2 and UPR pathways were interconnected as the inhibition of NRF2 reduced BiP protein levels. Mechanistically, the increased Ru-bdcurc-induced cell death, following NRF2 or BiP inhibition, correlated with the upregulation of the UPR apoptotic marker CHOP and with increased H2AX phosphorylation, a marker of DNA damage. The findings reveal that BiP and NRF2 interconnection was a key regulator of colon cancer cells resistance to Ru-bdcurc cytotoxic effect. Targeting that interconnection overcame the protective mechanism and enhanced the antitumor effect of the Ru-bdcurc compound.

## 1. Introduction

Colorectal cancer (CRC) is the second leading cause of cancer-related mortality worldwide [1,2]. Chemo- and targeted therapies offer only a limited increase in overall survival for these patients due to the acquired resistance to therapies, a major clinical issue in CRC [3]. The use of plant-derived natural compounds, often in combination with standard anticancer regimens [4], is now considered a valuable anticancer strategy to overcome drug resistance or re-sensitize chemoresistant cells, especially colon cancer cells, to reduce toxicity and to spare normal cells [5]. Among the natural compounds, curcumin shows a large variety of therapeutic properties in medical applications [6,7]. One obstacle to the use of curcumin in vivo though is its low absorption and poor bioavailability due to rapid metabolism, low water solubility, and stability [8]. To circumvent this problem, a number of strategies have been developed, involving the modification of its structure or application of drug systems delivery agents, such as nanoparticles, liposomes and micelles [9]. Another recent approach is based on the interaction of curcuminoid ligands with inorganic or organometallic moieties to provide more soluble and more assimilable systems [10,11,12].

Several cellular signaling pathways are dysregulated in CRC, including the unfolded protein response (UPR) and the antioxidant pathway, regulated by nuclear factor erythroid 2-related factor 2 (NRF2), leading to cancer onset, progression, and eventually chemoresistance [13,14]. The unfolded protein response (UPR) is a defense mechanism that cells adopt to cope with endoplasmic reticulum (ER) stress to restore homeostasis or activate cell death; therefore, its regulation can dictate the balance between cell survival and cell death [15,16]. Under severe and prolonged ER stress, the UPR activates pathways leading to cell death through the upregulation of C/EBP homologous protein (CHOP) [17]. The activation of the three UPR major sensors (namely ATF6α, IRE1 α and PERK) is controlled by the ER-resident chaperone molecule GRP78/BiP (glucose-regulated protein 78/binding immunoglobulin protein) [18]. BiP binds to proteins to stabilize and assist them in proper folding but may also promote cancer cell survival and chemoresistance due to its antiapoptotic property [19,20]. Thus, BiP is considered a good marker to predict the response to therapy and a promising target for anticancer therapies [21]. Hence, the inhibition of BiP increases the sensitivity of colon cancer cells to chemotherapy-induced apoptosis [22].

Nuclear factor erythroid 2-related factor 2 (NRF2) is a transcription factor that protects cells from oxidative stress. Under canonical activation by oxidative stress, NRF2 is released from the binding to its inhibitor Keap1, translocates into the nucleus, and induces the transcription of several target genes, including heme-oxygenase-1 (HO-1), glutathione reductase (Glut red), NAD(P)H quinone oxidoreductase 1 (NQO1), catalase, and superoxide dismutase (SOD) that help to restore the cellular redox homeostasis [23]. While NRF2 transient activation is considered to be mostly cytoprotective during the first phases of carcinogenesis, because it limits the DNA damage-induced DNA mutations, sustained NRF2 activation promotes cancer progression and chemoresistance because it counteracts the oxidative stress-induced cell death, particularly in response to therapies [23]. In this regard, the activation of the NRF2 target genes, such as HO-1 and NQO1, have been found to be involved in cancer progression. Thus, they can make cancer cells more resistant to anticancer agents, particularly to oxidative stress inducers [24,25]. Another NRF2 transcriptional target is p62/sequestome-1 (SQSTM1), an autophagy adaptor protein that has been shown to promote tumorigenesis [26]. P62/SQSTM1 stabilizes NRF2 in a non-canonical way by triggering Keap1 degradation, creating a positive feedback loop that sustains the antioxidant response that can help cancer cells survive stress [27,28,29]. Since NRF2 controls several pathways involved in tumor progression, its inhibition is considered a promising anticancer strategy to restore chemosensitivity [30].

Here we show that a cationic Ruthenium (Ru)(II)-cymeneene Bisdemethoxycurcumin complex (Ru-bdcurc) reduced colon cancer cells proliferation and induced cell death. At the molecular level, in response to Ru-bdcurc treatment, cancer cells activated NRF2 and BiP pathways whose pharmacologic inhibition increased Ru-bdcurc-induced cell death, suggesting that they acted as death resistant mechanisms to the drug. Therefore, the BiP/NRF2 axis can be considered a potential druggable target to increase the sensitivity of colon cancer cells to therapies.

## 2. Materials and Methods

### 2.1. Synthesis of [(cym)Ru(bdcurc)(PTA)]SO_3_CF_3_ Complex (Ru-bdcurc)

**Figure 1:**
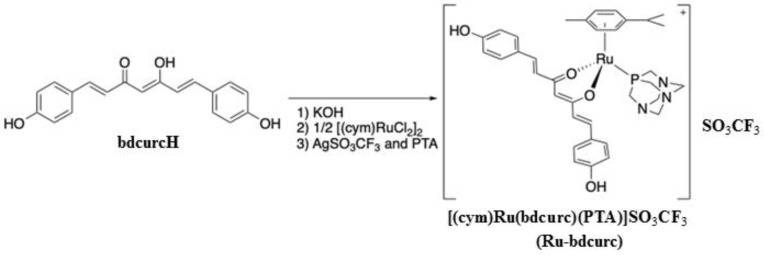
Molecular structure of the compound Ru-bdcurc.

The cationic Ruthenium (Ru)(II)−cymene complex containing bisdemethoxycurcumin and the hydrosoluble PTA phosphine ([(cym)Ru(bdcurc)(PTA)]SO_3_CF_3_) (where cym = cymene, bdcurc = bisdemethoxycurcumin and PTA = 1,3,5-triaza-7-phosphaadamantane) (herein Ru-bdcurc) (Figure 1) was synthesized by a procedure similar to that previously reported [31,32]. Briefly, [Ru(cymene)Cl_2_]_2_ (306 mg, 0.50 mmol) was added to a methanol solution (20 mL) of bisdemethoxycurcumin (bdcurcH, 308 mg, 1.00 mmol) and KOH (56 mg, 1.0 mmol) and the mixture was stirred under reflux for 24 h, and then AgSO_3_CF_3_ (257 mg, 1 mmol) was added to perform the metathesis reaction and replace the Cl in the ruthenium coordination sphere with the SO_3_CF_3_ anion. The reaction mixture was stirred for 1 h and filtered to remove AgCl. PTA (PTA = 1,3,5-triaza-7-phosphaadamantane; 157 mg, 1 mmol) was finally added to the filtrate, which was further stirred for 24 h at room temperature. The solvent was then removed, and the crude product was recrystallized from a 2/1 mixture of dichloromethane and n-hexane (25 mL) by cooling to 4 °C, slowly affording an orange crystalline powder (787 mg, 0.68 mmol, yield 68%), which was identified as the complex [(cym)Ru(bdcurc)(PTA)]SO_3_CF_3_.

The Ru-bdcurc complex is air stable and soluble in alcohols, acetone, acetonitrile, and DMSO and slightly soluble in chlorinated solvents.

Mp: 184−186 °C. Anal. Calcd for C_36_H_41_F_3_N_3_O_7_PRuS: C, 50.94; H, 4.87; N, 4.95. Found: C, 50.48; H, 4.73; N, 4.68. IR (cm^−1^): 3265br, 1620sh, 1602m, 1585sh, ν(C=C), 1270m, 1155s, 1025s ν(SO_3_CF_3_). ^1^H NMR (DMSO-*d*_6_, 293 K): δ 1.26 (d, 6H, CH_3_C_6_H_4_CH(C*H*_3_)_2_), 2.02 (s, 3H, C*H*_3_C_6_H_4_CH(CH_3_)_2_), 2.65 (m, 1H, CH_3_C_6_H_4_C*H*(CH_3_)_2_), 4.10 (sbr, 6H, PTA), 4.43 (sbr, 6H, PTA), 5.78 (s, 1H, C(1)H of bdcurc), 6.09 (dd, 4H, CH_3_C_6_*H*_4_CH(CH_3_)_2_), 6.65, (d, 2H, C(4,4′)H of bdcurc), 6.82 (d, 4H, C(6,6′)H and C(10,10′)H of bdcurc), 7.37 (d, 2H, C(3,3′)H of bdcurc), 7.51 (d, 4H, C(7,7′)H and C(9,9′)H of bdcurc), 10.00 (sbr, 2H, OH). ^13^C NMR (DMSO-*d*_6_, 293 K): δ 16.7 (s, CH_3_C_6_H_4_CH(*C*H_3_)_2_), 22.2 (s, *C*H_3_C_6_H_4_CH(CH_3_)_2_), 30.4 (s, CH_3_C_6_H_4_*C*H(CH_3_)_2_), 51.1 (d, P*C*H_2_N, ^1^*J*(C-P) = 12.7 Hz, PTA), 72.2 (d, N*C*H_2_N, ^3^*J*(C-P) = 7.2 Hz, PTA), 88.5, 90.1, 96.4, 103.8 (s, CH_3_*C*_6_H_4_CH(CH_3_)_2_), 104.7 (s, *C*(1,1′) of bdcurc), 116.4 (s, *C*(9,9′) and *C*(7,7′) of bdcurc), 123.6 (s, *C*(10,10′) and *C*(6,6′) of bdcurc), 126.5 (s, *C*(5,5′) of bdcurc), 130.5 (s, *C*(3,3′) of bdcurc), 140.0 (s, *C*(4,4′) of bdcurc), 160.0 (s, *C*(8,8′) of bdcurc), 180.2 (s, *C*(2,2′)=O of bdcurc). ^31^P NMR (DMSO-*d*_6_, 293 K): δ −27.1. ESI-MS (+) CH_3_OH (*m*/*z* [relative intensity, %]): 700 [100] [Ru(cymene)(bdcurc)(PTA)]^+^, 543 [20] [Ru(cymene)(bdcurc)]^+^. Λ_m_ (CH_3_OH, 298 K, 10^−3^ mol/L): 90 S cm^2^ mol^−1^. Λ_m_ ((CH_3_)_2_SO, 298 K, 10^−3^ mol/L): 42 S cm^2^ mol^−1^. UV-Visible spectrum (DMSO, l_max_(nm)): 253 (11487 *e* M^−1^ cm^−1^, n → π*), 417 (7731 *e* M^−1^ cm^−1^, p → p*), 480sh (4153 *e* M^−1^ cm^−1^, MLCT Ru(4d^6^) → π*). UV-Visible spectrum (EtOH, l_max_(nm)): 234 (11,603 *e* M^−1^ cm^−1^, n → π*), 413 (7669 *e* M^−1^ cm^−1^, *π* → π*), 468sh (5113 *e* M^−1^ cm^−1^, MLCT Ru(4d^6^) → π*).

Its analytical and spectroscopic characterization confirmed the cationic structure shown in Figure 1. The electronic spectra recorded in DMSO and EtOH displayed two intense bands at 234–253 nm and 413–417 nm, characteristic of n−π* and π−π* transitions of the bdcurc ligand, while the shoulder at 468–480 nm is ascribed to MLCT (metal-ligand charge transfer) from the filled 4d orbitals of ruthenium to the empty π* orbital of the ligand bdcurc. The NMR spectra are shown in Supplementary Figure S1.

Its stability toward hydrolysis was previously investigated by ^31^P NMR spectroscopy under pseudo-pharmacological conditions at 37 °C (5 mM NaCl solution in D_2_O containing 10% [D_6_]DMSO, corresponding to the low intracellular NaCl concentration in cells, and in 100 mM NaCl solution in D_2_O containing 10% [D_6_]DMSO, approximating to the higher NaCl levels in blood plasma); within 24 h in the 100 mM aqueous NaCl solution, Ru-brdcurc decomposes into the well-known complex [Ru(cymene)(PTA)Cl_2_] [33], whereas in the 5 mM aqueous NaCl solution the complex is stable.

The Ru-bdcurc complex was dissolved in DMSO and stored at −20 before using it at different concentrations.

### 2.2. Cell Culture and Reagents

Human colon cancer HCT116 (kindly provided by Prof. Ber Vogelstein, Johns Hopkins University, Baltimore, MD, USA) and RKO cells were maintained in Dulbecco’s modified Eagle’s medium (DMEM) (Life Technologies-Invitrogen, Eggenstein, Germany), supplemented with 10% heat-inactivated fetal bovine serum (FBS) (Corning Life Sciences, New York, NY, USA), plus glutamine and antibiotics (Corning Life Sciences, New York, NY, USA) in a humidified atmosphere with 5% CO_2_ at 37 °C. Cells underwent routine testing to ensure that they were mycoplasm negative. The inhibitor of the antioxidant response Brusatol (Sigma-Aldrich, St Louis, MO, USA) [34,35] was used at 100 µM for 4 h pre-treatment, as previously reported [36]; Bip/GRP78 inhibitor HA15 (Sigma-Aldrich, St Louis, MO, USA, SML2118) [37,38] was used at 10 µM for 1 h pre-treatment, as previously reported [20].

### 2.3. Cell Viability Assay

Cells were plated in six-well plates and treated the day after with a different concentration (50, 100 µM) of Ru-bdcurc for 24, 48, and 72 h or in combinations with a 4 h pre-treatment of brusatol (100 µM) or 1 h pre-treatment of HA15 (10 µM), as indicated. After treatments, both floating and adherent cells were collected and subjected to Trypan blue staining (Sigma-Aldrich, St Louis, MO, USA, #72571). Cell viability of triplicates was assessed by counting blue (dead)/total cells with a Neubauer hemocytometer using light microscopy.

### 2.4. Proliferation Assay (XTT)

Cell proliferation was evaluated by XTT assay using the Cell Proliferation II kit following the manufacturer’s instructions (Roche Diagnostic S.p.A., Monza, Italy), as previously reported [39]. Briefly, cells were seeded in 96-well culture plates (5 × 10(3) cells/well, in triplicates) and were treated the day after with Ru-bdcurc (100 µM) alone or in combination with a 4 h pre-treatment of brusatol (100 µM) for the indicated time. After treatment, XTT was added for 4 h at 37 °C before stopping the formazan formation with the solubilization solution. The absorbance was measured at a wavelength of 492 nm, using the Multiskan FC microplate reader, (Thermo Fisher Scientific, Walthman, MA, USA).

### 2.5. Western Blotting

Western blotting was performed, as previously reported [40]. Briefly, cells were lysed in lysis buffer (50 mM Tris–HCl, pH 7.5, 150 mM NaCl, 5 mM EDTA, 150 mM KCl, 1 mM dithiothreitol and 1% Nonidet P-40) (all from Sigma- Aldrich, Dorset, UK) containing protease inhibitors (CompleteTM, Mini Protease Inhibitor Cocktail, Merck, Life Science S.r.l., Milan, Italy). Proteins were separated by loading 10–30 ug of total cell lysates on denaturing 8–15% SDS-PAGE (polyacrylamide gel electrophoresis) gels (Bio-Rad, Hercules, CA, USA), following semidry blotting to polyvinylidene difluoride (PVDF) membranes (Immobilon-P, Merk-Millipore, Milan, Italy). Unspecific signals were blocked by incubating the membranes in Tris buffered saline containing 0.1% Tween 20 (TBS) and 3% BSA (Sigma-Aldrich, Dorset, UK) for 1 h. Membranes were then probed with the primary antibodies and subsequently with the appropriate secondary antibodies coupled to horseradish peroxidase (HRP) (Bio-Rad, Hercules, CA, USA). The enzymatic signal was visualized by chemiluminescence (ECL Detection system, Amersham GE Healthcare, Milan, Italy). Densitometry was performed on ECL results with ImageJ software (1.47 version, NIH, Bethesda, MD, USA) which was downloaded from the NIH website (http://imagej.nih.gov/ij, accessed on 1 August 2022) and the relative band intensity was normalized to β-actin signals and plotted as protein expression/β-actin ratio.

### 2.6. Antibodies

To detect the protein expression on Western blot membranes, the following antibodies were used: mouse monoclonal anti-p62/SQSTM1 (D-3, sc-28359) (1:1000), mouse monoclonal anti-HO-1 (A-3, sc-136960) (1:1000) and mouse monoclonal anti-NQO1/A180, sc-32793) (Santa Cruz Biotechnology Inc, Dallas, TX, USA), rabbit polyclonal anti-NRF2 (1:1000) (Abcam, Cambridge, UK, #ab62352), mouse monoclonal anti-phospho-Histone H2AX (Ser139 clone JBW301) (1:1000) (Sigma-Aldrich, St Louis, MO, USA, #05-636), rabbit polyclonal anti-CHOP (GADD153) (1:1000) (Proteintech, Rosemont, IL; USA, #15204-1-AP), and rabbit polyclonal anti-BiP/GRP78 (1:5000) (Proteintech,Rosemont, USA, #11587-1-AP). Mouse monoclonal β-actin (Ab-1) (1:10,000) (Calbiochem, San Diego, CA, USA, #CP01), was used as protein loading control.

### 2.7. RNA Extraction and Semiquantitative Reverse Transcription (RT)-Polymerase Chain Reaction (PCR) Analysis

RT-PCR analysis was performed, as previously reported [40]. Briefly, total RNA extraction was performed by using TRIzol Reagent (Thermo Fisher Scientific, Walthman, MA, USA); cDNA was synthesized by using an MuLV reverse transcriptase kit (Applied Biosystems, Foster City, CA, USA); semiquantitative reverse-transcribed (RT)-PCR was carried out with 2 μL cDNA reaction and genes specific oligonucleotides under conditions of linear amplification by using Hot-Master Taq polymerase (Thermo Fisher Scientific, Walthman, MA, USA). PCR products were run on a 2% agarose gel and visualized with GelRed Nucleic Acid gel stain (Biotium, San Francisco, CA, USA). The housekeeping 28S gene, used as the internal standard, was amplified from the same cDNA reaction mixture. Densitometric analysis was applied to quantify mRNA levels compared to control gene expression.

### 2.8. Statistical Analysis

The results are expressed as mean ± standard deviation (S.D.) of at least three independent experiments. A two-tailed Student’s *t*-test was applied for two-samples comparison. A difference was considered statistically significant when the *p*-value was at least ≤0.05.

## 3. Results

### 3.1. Ru-bdcurc Compound Induces Cell Death in Colon Cancer Cells

**Figure 2:**
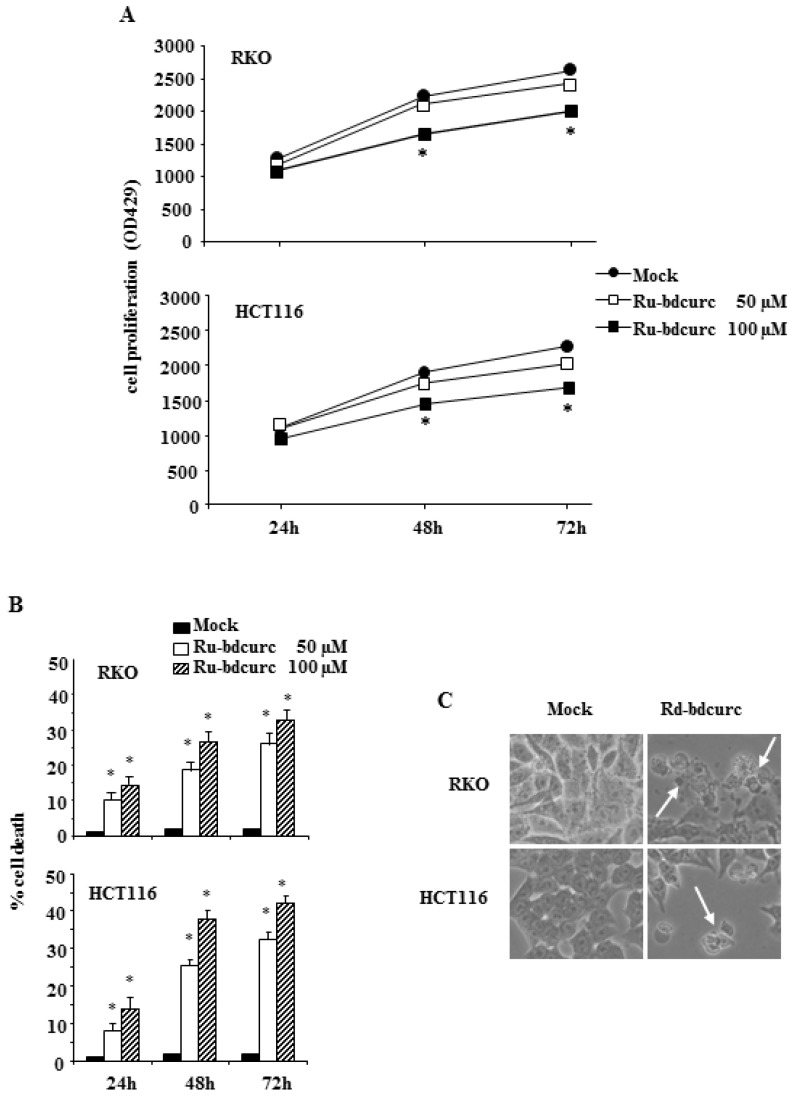
Dose-and time-dependent inhibition of colon cancer cells proliferation by Ru-bdcurc. (**A**) RKO and HCT116 colon cancer cells were left untreated or treated with different doses of Ru-bdcurc (50 and 100 µM) for 24, 48, and 72 h before cell proliferation was measured by XTT assay. (**B**) Cell viability of cells treated as in (**A**) was measured by Trypan blue exclusion assay and (**C**) live cell images were taken by light microscopy. * *p* ≤ 0.05. White arrows indicate dead cells.

RKO and HCT-116 cells were treated with Ru-bdcurc compound (50 and 100 μM) for 24, 48, and 72 h, and then cell proliferation was measured using the XTT assay. The data show that Ru-bdcurc treatments inhibited cell proliferation of both cell lines in a time-dependent fashion and in a dose-dependent manner (Figure 2A). In agreement, Ru-bdcurc treatments induced both RKO and HCT-116 cell death in a dose- and time-dependent manner (Figure 2B). Cell death was also evidenced microscopically where distinct signs of cell shrinkage were observed (Figure 2C).

These results indicate that the Ru-bdcurc complex was able to trigger colon cancer cells death.

### 3.2. Induction of NRF2 and BiP Expression in Response to Rubdcurc Treatment

**Figure 3:**
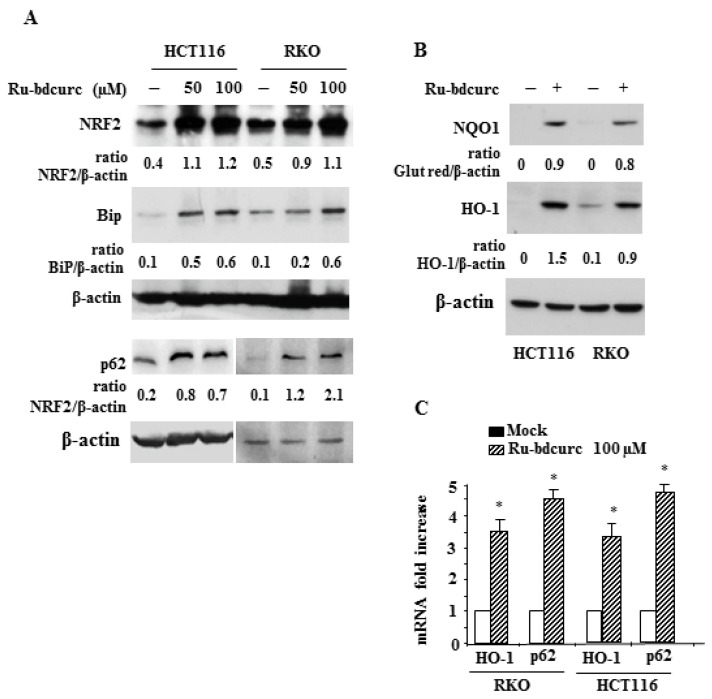
Activation of NRF2 and BiP pathways in response to Ru-bdcurc treatment. (**A**) Western blot analysis of the indicated proteins in RKO and HCT116 colon cancer cells treated with 50 and 100 µM Ru-bdcurc for 48 h or (**B**) with 100 µM Ru-bdcurc for 48 h. β-actin was used as protein loading control. The ratio of the proteins level vs. β-actin, following densitometric analysis, is reported. (**B**) Total mRNA was extracted from cells treated as in (A) to evaluate HO-1 and p62 gene expression by RT-PCR of reverse transcribed cDNA. Histograms represent the mean of three independent experiments ± S.D. (**C**) Western blot analysis of p62 in RKO and HCT116 cells treated as in (**A**) with 50 and 100 µM Ru-bdcurc for 48 h. The ratio of the proteins level vs. β-actin, following densitometric analysis, is reported. * *p* ≤ 0.05.

Although cancer cells underwent cell death, some cells appeared to be less sensitive to the cytotoxic effect of Ru-bdcurc. Therefore, we investigated the possible mechanisms of death resistance. As shown in Figure 3, following Ru-bdcurc treatment, cancer cells greatly increased the NRF2 protein levels and the expression of NRF2 targets, including HO-1, NQO1, and p62/SQSTM1 (Figure 3A–C), suggesting the induction of NRF2 transcriptional activity. In addition, cancer cells also increased the protein levels of BiP in response to Ru-bdcurc treatment (Figure 3A).

Bip is a molecule belonging to the UPR pathway that has been shown to promote cancer cell survival and chemoresistance due to its antiapoptotic property [19,20,41]. Altogether, these results indicate a concomitant activation of the NRF2 and UPR pathways, potentially acting to counteract the Ru-bdcurc cytotoxic effect.

### 3.3. NRF2 and BiP Pathways as Death Resistant Mechanisms to Ru-bdcurc Cytotocicity

**Figure 4:**
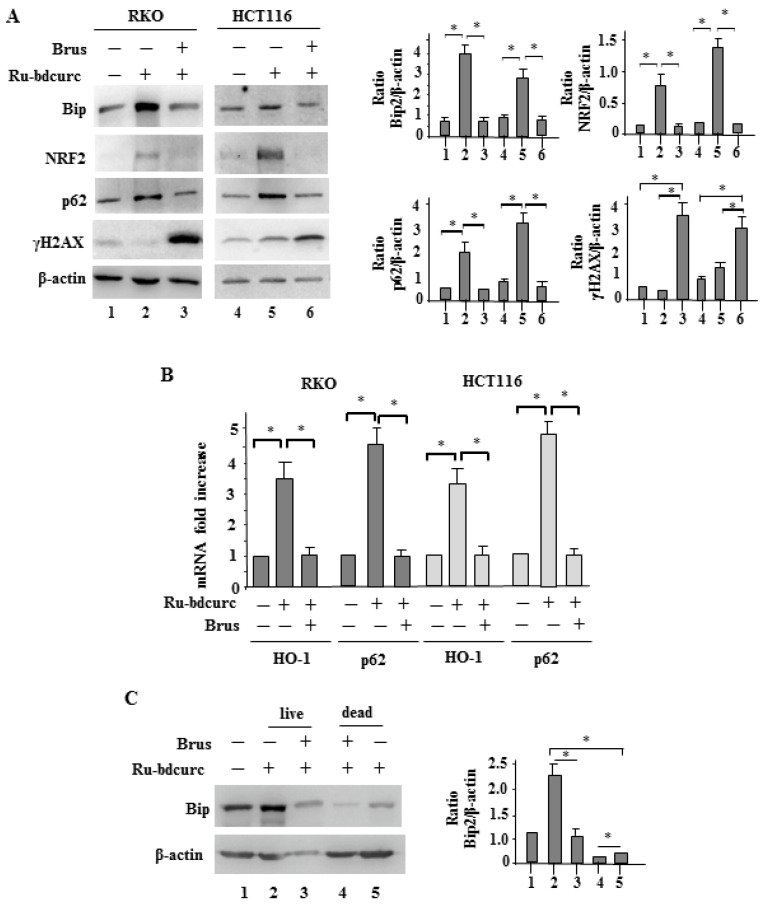
Inhibition of NRF2 reduced BiP and induced DNA damage in response to Ru-bdcurc. (**A**) Western blot analysis of the indicated proteins in RKO and HCT116 colon cancer cells pre-treated with 100 brusatol (Brus) for 4 h and the treated with 100 µM Ru-bdcurc for 24 h. β-actin was used as protein loading control. The ratio of the proteins level vs. β-actin, following densitometric analysis, is reported in the right panels. (**B**) Total mRNA was extracted from cells treated as in (**A**) to evaluate *HO-1* and *p62* gene expression by RT-PCR of reverse transcribed cDNA. Histograms represent the mean of three independent experiments ± S.D. (**C**) RKO and HCT116 cells were pre-treated with 100 brusatol (Brus) for 4 h and then treated with 100 µM Ru-bdcurc for 48 h. After treatment, the expression of BiP in living or dead cells was evaluated by Western blot analysis. β-actin was used as protein loading control. The ratio of BiP level vs. β-actin, following densitometric analysis, is reported in the right panel. * *p* ≤ 0.05.

In order to evaluate the role of NRF2 on the Ru-bdcurc cytotoxic effect, we pharmacologically inhibited it with brusatol [34,35]. We found that brusatol co-treatment reduced the Ru-bdcurc-induced protein levels of NRF2 and its activity, as assessed by the downregulation of the NRF2 targets p62/SQSTM1 and HO-1 (Figure 4A,B). Of note, the brusatol/Ru-bdcurc co-treatment greatly induced γH2AX levels (Figure 4A), compared to the single treatment, suggesting that the inhibition of the NRF2 antioxidant activity correlated with increased DNA damage following Ru-bdcurc treatment. Thus, H2AX phosphorylation, generating γH2AX, is considered a marker of DNA damage [42,43]. Interestingly, brusatol co-treatment significantly reduced the Ru-bdcurc-induced BiP expression levels (Figure 4A).

To evaluate the interplay between NRF2 and BiP, we treated cells with Ru-bdcurc and then isolated the cell fraction that remained attached to the bottom of the wells (live cells, resistant to the drug cytotoxic effect) and the cell fraction floating in suspension (dead cells, sensitive to the drug cytotoxic effect). The results show that the Ru-bdcurc-induced BiP protein levels were more expressed in the population of attached live cells compared to the population of floating dead cells (Figure 4C, compare lane 2 with lane 5). Brusatol co-treatment strongly reduced the Ru-bdcurc-induced BiP protein levels in the population of attached live cells (Figure 4C, compare lane 2 with lane 3) and further reduced the BiP level in the floating dead cells (Figure 4C, compare lane 5 with lane 4). This result suggests that inhibiting NRF2 also inhibits BiP and that the occurrence of NRF2 and BiP interplay, mainly in the attached live cells, was acting as a death resistant mechanism to the Ru-bdcurc cytotoxic effect.

**Figure 5:**
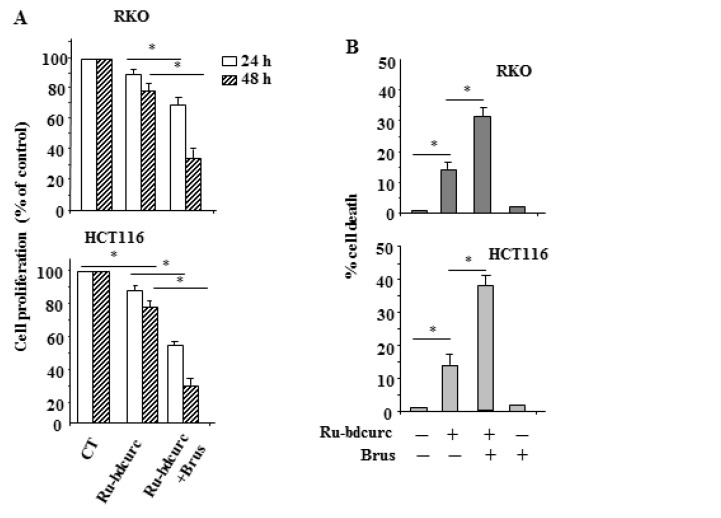

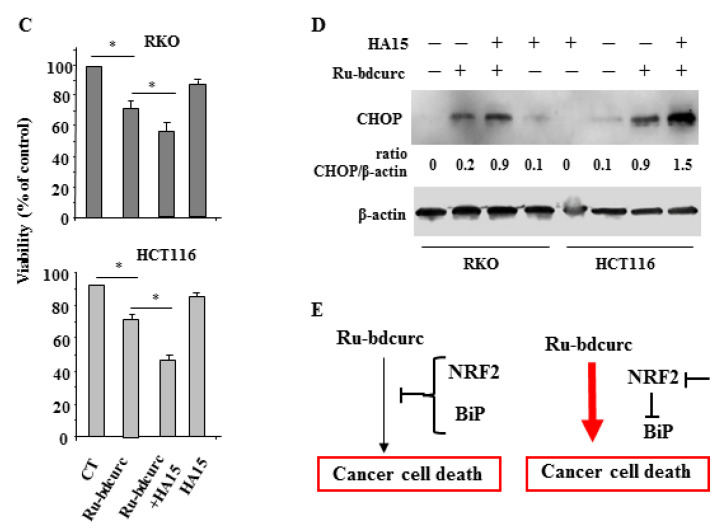
Inhibition of NRF2 or BiP increases the Ru-bdcurc cytotoxic effect in colon cancer cells. (**A**) RKO and HCT116 colon cancer cells were left untreated or pre-treated with 100 µM brusatol (Brus) for 4 h and then treated with 100 µM Ru-bdcurc for 24 and 48 h, before cell proliferation was measured by XTT assay. (**B**) Cell viability of RKO and HCT116 cells pre-treated with 100 µM brusatol (Brus) for 4 h and then treated with 100 µM Ru-bdcurc for 24 was measured by Trypan blue exclusion assay. (**C**) Cell viability of RKO and HCT116 cells, pre-treated with 10 µM HA15 for 1 h and then treated with 100 µM Ru-bdcurc for 48 h, was measured by Trypan blue exclusion assay. * *p* ≤ 0.05. (**D**) Western blot analysis of CHOP in RKO and HCT116 colon cancer cells pre-treated with 10 µM HA15 for 1h and the treated with 100 µM Ru-bdcurc for 48 h. β-actin was used as protein loading control. The ratio of the proteins level vs. β-actin, following densitometric analysis, is reported in the right panels. (**E**) Schematic summary of the negative effect of NRF2 and BiP pathways on Ru-bdcurd cytotoxic effect (left panel); inhibition of NRF2 also inhibits BiP and improves the Ru-bdcurc-induced cell death (right panel).

Finally, we correlated the biochemical results to the biological outcome. We found that the pharmacologic inhibition of NRF2 with brusatol significantly improved the Ru-bdcurc-induced inhibition of cell proliferation, as assessed by XTT assay (Figure 5A) and, in agreement, potentiated the cell death induced by Ru-bdcurc (Figure 5B). These findings confirm that the NRF2 pathway was indeed acting as a mechanism of resistance to the Ru-bdcurc-induced cell death. We finally investigated the role of BiP, in this setting, by pharmacologic inhibition with HA15 [37,38]. We found that BiP inhibition potentiated the Ru-bdcurc-induced cell death (Figure 5C). The increased cytotoxicity following HA15 co-treatment correlated with a greater increase of CHOP and γH2AX protein levels (Figure 5D), highlighting the antiapoptotic role of BiP in this setting and the strict interconnection between a strong ER stress and the occurrence of DNA damage.

Altogether, these results indicate that the inhibition of the NRF2 or BiP pathways potentiated the pro-death effect of UPR and the cytotoxic effect of Ru-bdcurc.

## 4. Discussion

In this study we evaluated the anticancer effect of an organometallic cationic Ruthenium (Ru)(II)−cymene complex containing bisdemethoxycurcumin (Ru-bdcurc). We found that the compound-mediated cytotoxic effect against colon cancer cells was greatly improved by the concomitant inhibition of NRF2 and BiP, highlighting a critical interplay between these two molecules and their pathways that cancer cells exploit to resist to the cytotoxic effect of the anticancer therapies.

Sustained NRF2 activation protects cancer cells against chemo- and radiation therapies, promoting molecular pathways that support cell proliferation [23]. This outcome is achieved not only by the antioxidant effect of NRF2, but also because NRF2 collaborates with several different oncogenic pathways, sometime interconnected and/or that act in a feedback loop with NRF2, leading cancer cells to adapt to stresses induced, for instance, by anticancer therapies [44,45]. One of these oncogenes is p62/SQSTM1 [26] that is a target of NRF2 and can stabilize NRF2 in a non-canonical way by triggering Keap1 degradation [27]. Moreover, the NRF2/p62 interplay creates a positive feedback loop that has been reported to help cancer cells survive in conditions of stress, therefore promoting chemoresistance [28]. Therefore, the interplay between NRF2 and p62/SQSTM1 is considered a potential target to be exploited for anticancer therapeutical benefits. Here, we found that p62/SQSTM1 was induced in response to Ru-bdcurc and that this induction was counteracted by the inhibition of NRF2 activity. The inhibition of NRF2/p62/SQSTM1 crosstalk correlated with an increased cell death response to the Ru-bdcurc treatment, confirming that the NRF2/p62/SQSTM1 axis was acting as a pro-survival pathway.

High levels of NRF2 have been found in colon cancer patients and are associated with poor prognosis and resistance to therapies [14,46,47]. NRF2 inhibition is indeed considered a potential anticancer strategy to restore chemosensitivity [30], as also demonstrated by our previous studies [39,40,48,49,50]. One of the main effectors of NRF2-dependent cell response that contributes to survival advantage, tumor aggressiveness, chemoresistance, and poor patient outcome is HO-1 [24,51]. In healthy cells, HO-1 plays a key role in maintaining redox homeostasis; however, many studies demonstrated its tumorigenic role in cancer proliferation and resistance to therapies in different tumor types [52]. HO-1 is therefore a potential biomarker for cancer progression and a promising target to improve the anticancer therapies. In line with this issue, here we found that the induction of HO-1 in response to NRF2 activation was counteracted by inhibiting NRF2, and that the inhibition of the NRF2 pathway increased the cytotoxic effect of Ru-bdcurc treatment.

Here we also found that the increased Ru-bdcurc-induced cell death following NRF2 inhibition correlated with high levels of H2AX phosphorylation, an early cellular response to the induction of the DNA double-strand breaks, that is considered a marker of DNA damage [42]. H2AX phosphorylation has been shown to mediate apoptosis and its inhibition has been shown to correlate with tumor resistance to radio-and chemotherapies [53]. Our findings highlight the NRF2 cytoprotective effect, likely by its antioxidant function, to counteract the DNA damage-induced cell death in response to Ru-bdcurc treatment. Indeed, increased NRF2 activity has been shown to reduce the oxidation-mediated DNA damage by, for instance, ionizing radiation or cisplatin treatment [54,55].

Another molecule that can promote cancer cell survival and chemoresistance due to its antiapoptotic property is BiP [19,20,21,41]. Here we found that in response to Ru-bdcurc treatment, colon cancer cells increased the levels of BiP. Interestingly, we found that BiP levels were mainly increased in the fraction of cells resistant to Ru-bdcurc-induced cell death, and that BiP inhibition improved the compound cytotoxic effect. This is in line with studies showing that the inhibition of BiP increases the sensitivity of colon cancer cells to chemotherapy-induced cell death [22]. The increased Ru-bdcurc-induced cell death after BiP inhibition correlated with the upregulation of CHOP, the UPR apoptotic marker activated under sustained and chronic conditions of ER stress [13]. We also found a link between NRF2 and UPR. Thus, the BiP downregulation was observed following NRF2 inhibition with brusatol, suggesting that its inhibition could be the additional mechanism, other than the downregulation of the NRF2 pathway, through which brusatol increased the Ru-bdcurc-induced cell death, as the specific BiP targeting by HA15, in comparison, induced the same effect. However, the molecular mechanisms of NRF2 and UPR that interplay in this setting need to be evaluated further, as it has been reported that NRF2 can be activated in the course of ER stress by IRE1α and PERK branches of UPR [56,57]. Our results are in agreement with the finding that both NRF2 and BiP are adaptive response pathways that cancer cells overexpress in order to survive stress, and in particular chemotherapeutic agents [15,17,18,21,22,58,59]. The overexpression of those adaptive pathways, however, turns out to be an Achille’s heel for cancer cells [60]. Thus, targeting NRF2 and/or BiP induces cancer cell death by concomitantly exacerbating ER stress and inducing DNA damage, as also demonstrated in this study. The NRF2/BiP interplay may be considered a promising target in therapeutical anticancer strategies to overcome pro-survival mechanisms that favor cancer progression and chemoresistance [61,62].

The use of plant-derived natural compounds [4,63], often in combination with standard anticancer regimens, is now considered a valuable anticancer strategy to overcome drug resistance or to re-sensitize chemoresistant cells [5]. Natural compounds, such as curcumin, have been extensively evaluated in preclinical studies in the last years for their anti-cancer and anti-inflammatory properties, and for their low toxicity [64]. Curcumin is the major biologically active polyphenolic constituent in turmeric plant (*Curcuma longa*), along with two other curcuminoids that occur in lesser amounts, namely, demethoxycurcumin (dcurcH) and bis-demethoxycurcumin (bdcurcH), that also present a large variety of therapeutic properties in medical applications [6,7,8,9,10,11]. One obstacle to the use of curcumin in vivo is its low absorption and poor bioavailability due to rapid metabolism, low water solubility, and stability [8]. To circumvent this problem, a number of strategies have been developed, involving the modification of its structure or application of drug systems delivery agents, such as nanoparticles, liposomes, and micelles [9]. Another recent approach is based on the interaction of curcuminoid ligands with inorganic or organometallic moieties to provide more soluble and more assimilable systems [10,12]. Organometallic ruthenium(II) cymene complexes have been shown to possess promising pharmacological properties [65]. The relatively robust nature of ruthenium(II)-cymene complexes allows their rational modification such that an organic compound of known therapeutic value may be tethered to the motif [66]. It was previously shown that organometallic ruthenium(II) complexes with a PTA ligand display antimetastatic activity in vivo, [33] as well as an intrinsic antiangiogenic activity [67] and the ability to reduce the growth of certain primary tumors [68]. Here, we show that the Ru-bdrcurc presented anticancer activity, in agreement with several (arene)Ru(II) complexes containing curcuminoid ligands, that we previously developed [31,32,50,69,70,71]. Moreover, Ru-brdcurc could be a potential anticancer molecule to also be used efficiently in vivo, not only for its good water solubility and stability but also because it has been proven to spare nontumorous cells [31].

In summary, the results of this study indicate that BiP and NRF2 were interconnected pathways and contributed to colon cancer cells resistance to Ru-bdcurc cytotoxic effect. Therefore, this interconnection could be considered a potential druggable target for the development of more efficient therapies against colon cancer in combination with organometallic ruthenium (Ru)(II)-cymene complexes.

## Data Availability

The datasets generated and analyzed during the current study are available from the corresponding authors upon reasonable request.

## Supplementary Materials

The following supporting information can be downloaded at: https://www.mdpi.com/article/10.3390/biomedicines11020593/s1, Figure S1: NMR spectra.
