# Supplementary material for: NRF2 and Bip Interconnection Mediates Resistance to the Organometallic Ruthenium-Cymene Bisdemethoxycurcumin Complex Cytotoxicity in Colon Cancer Cells
Source: Biomedicines. 2023 Feb 16;11(2):593. doi: 10.3390/biomedicines11020593 (PMC9953010; doi:10.3390/biomedicines11020593)
Supplement: Supplementary file 1 [file biomedicines-11-00593-s001.zip › biomedicines-2206995-supplementary.pdf]

Supplementary Figure S1A,B,C: NMR spectra

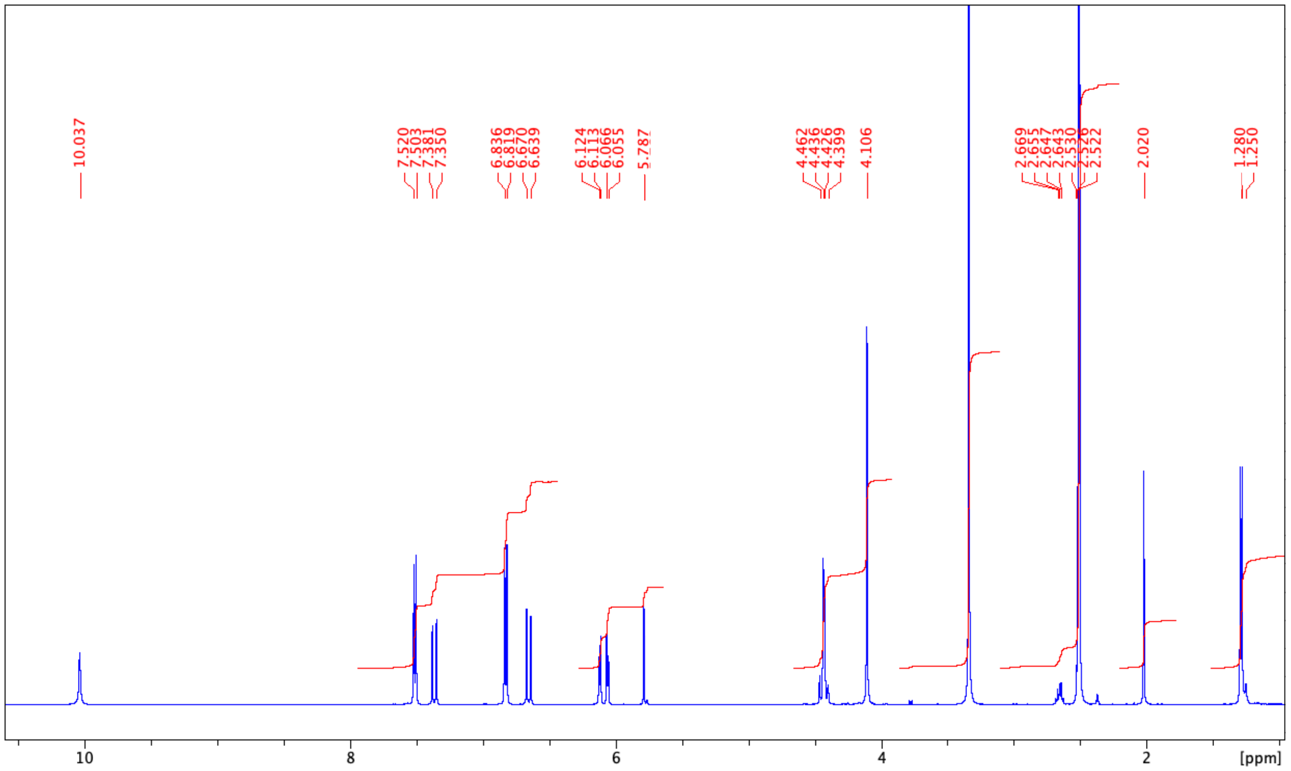

Figure S1A. ^{1}H NMR of Ru-bdcurc in DMSO-d_6 at 293 K.

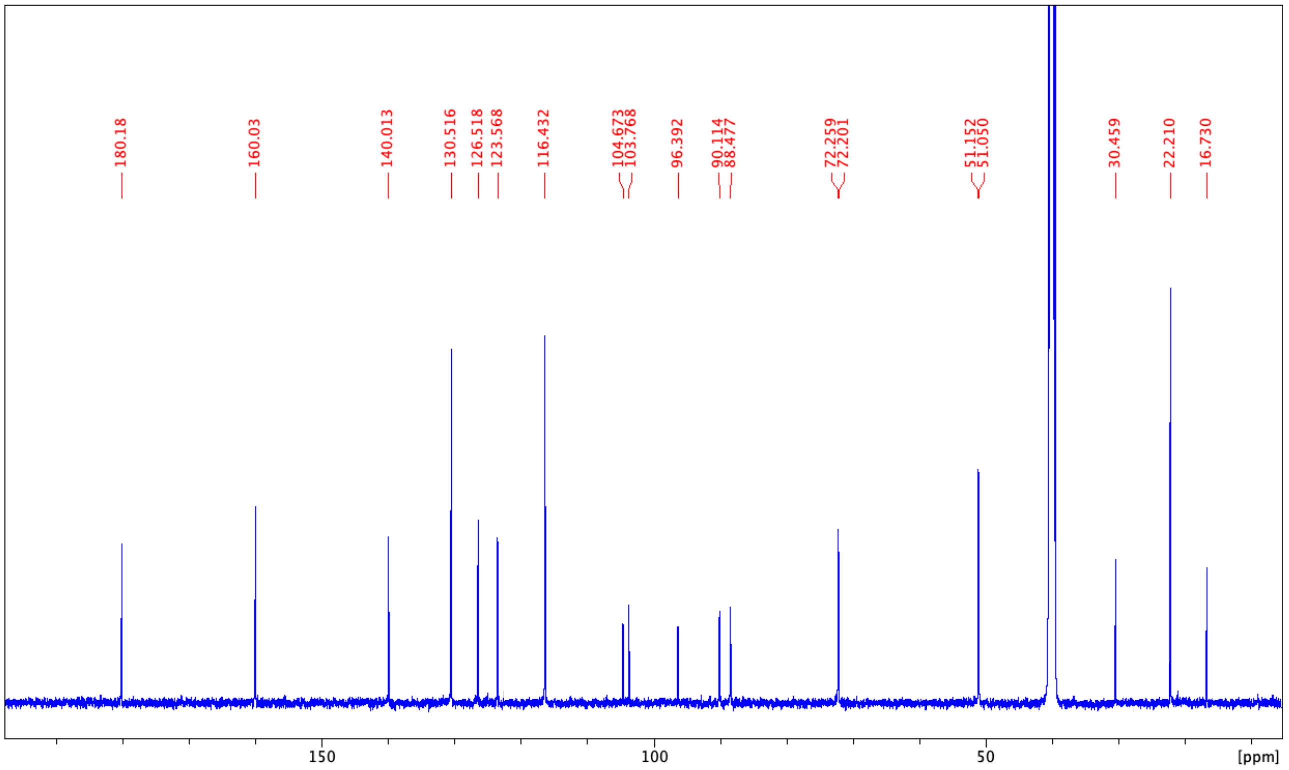

Figure S1B. $^{13}C\{^{1}H\}$ NMR of Ru-bdcurc in DMSO-d_6 at 293 K.

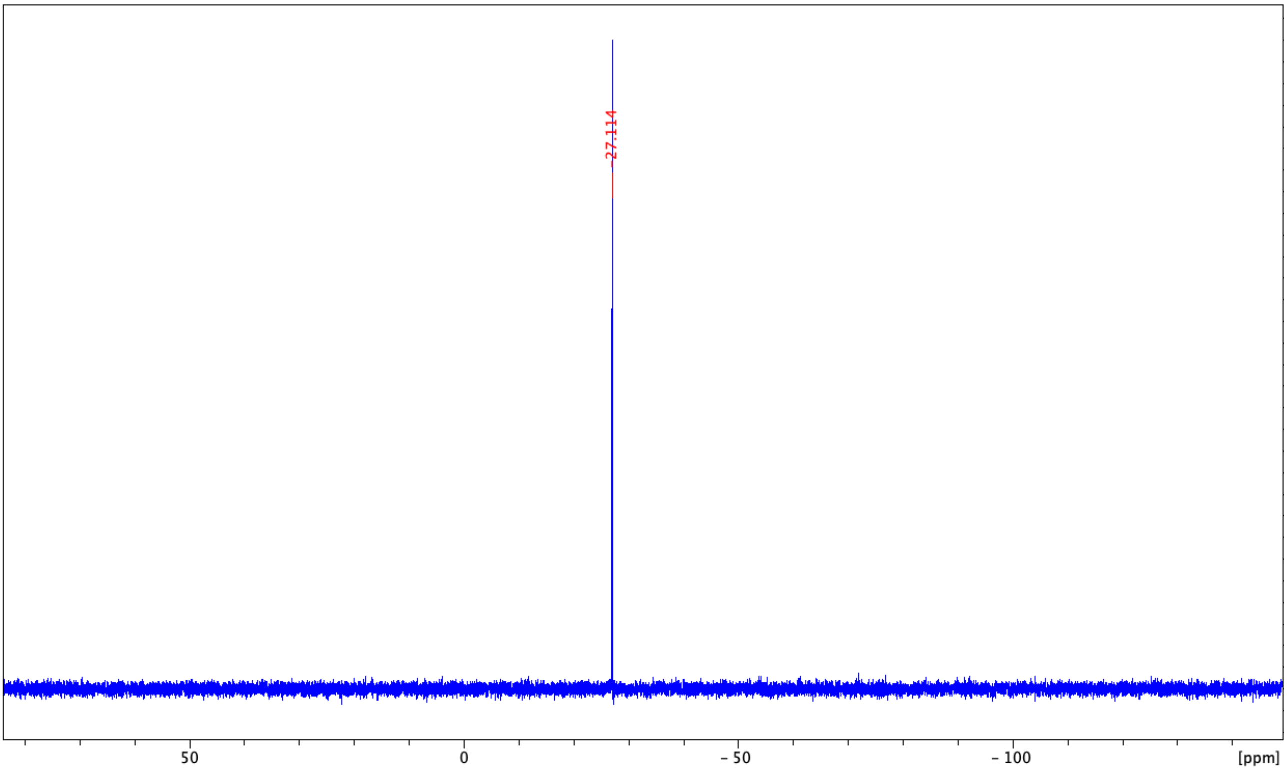

Figure S1C. $^{31}P\{^{1}H\}$ NMR of Ru-bdcurc in DMSO-d_6 at 293 K.
